# Case Report: Preemptive Treatment With Low-Dose PD-1 Blockade and Azacitidine for Molecular Relapsed Acute Myeloid Leukemia With RUNX1-RUNX1T1 After Allogeneic Hematopoietic Stem Cell Transplantation

**DOI:** 10.3389/fimmu.2022.810284

**Published:** 2022-02-02

**Authors:** Yutong Tang, Zhenyang Zhou, Han Yan, Yong You

**Affiliations:** ^1^ Institute of Hematology, Union Hospital, Tongji Medical College, Huazhong University of Science and Technology, Wuhan, China; ^2^ Department of Radiology, Union Hospital, Tongji Medical College, Huazhong University of Science and Technology, Wuhan, China; ^3^ Hubei Province Key Laboratory of Molecular Imaging, Wuhan, China

**Keywords:** acute myeloid leukemia, allogeneic hematopoietic stem cell transplantation, immune checkpoint inhibitors, preemptive treatment, RUNX1-RUNX1T1, hypomethylating agents, graft-versus-host disease, graft-versus-leukemia

## Abstract

Acute myeloid leukemia (AML) patients who develop hematological relapse (HR) after allogeneic hematopoietic stem cell transplantation (allo-HSCT) generally have dismal clinical outcomes. Measurable residual disease (MRD)-directed preemptive interventions are effective approaches to prevent disease progression and improve prognosis for molecular relapsed patients with warning signs of impending HR. In this situation, boosting the graft-vs-leukemia (GVL) effect with immune checkpoint inhibitors (ICIs) might be a promising prevention strategy, despite the potential for causing severe graft-vs-host disease (GVHD). In the present study, we reported for the first time an AML patient with *RUNX1-RUNX1T1* who underwent preemptive treatment with the combined application of tislelizumab (an anti-PD-1 antibody) and azacitidine to avoid HR following allo-HSCT. On day +81, molecular relapse with MRD depicted by *RUNX1-RUN1T1*-positivity as well as mixed donor chimerism occurred in the patient. On day +95, with no signs of GVHD and an excellent eastern cooperative oncology group performance status (ECOG PS), the patient thus was administered with 100 mg of tislelizumab on day 1 and 100 mg of azacitidine on days 1-7. After the combination therapy, complete remission was successfully achieved with significant improvement in hematologic response, and the MRD marker *RUNX1-RUNX1T1* turned negative, along with a complete donor chimerism in bone marrow. Meanwhile, the patient experienced moderate GVHD and immune-related adverse events (irAEs), successively involving the lung, liver, lower digestive tract and urinary system, which were well controlled by immunosuppressive therapies. As far as we know, this case is the first one to report the use of tislelizumab in combination with azacitidine to prevent post-transplant relapse in AML. In summary, the application of ICIs in MRD positive patients might be an attractive strategy for immune modulation in the future to reduce the incidence of HR in the post-transplant setting, but safer clinical application schedules need to be explored.

## Introduction

For patients who suffer from intermediate or high-risk acute myeloid leukemia (AML), allogeneic hematopoietic stem cell transplantation (allo-HSCT) represents a major curative approach principally through the induction of the graft-vs-leukemia (GVL) effect (1–3). However, recurrence remains the most frequent cause of treatment failure as well as post-transplantation mortality, with up to 50% of AML patients eventually relapsing after allo-HSCT (4–6). Prognosis in patients with hematological relapse (HR) is generally dismal, and treatment options are often limited due to the depressing outcomes in terms of tolerability and efficacy (7–9). Therefore, the prevention of HR is still the primary goal in the care of post-HSCT relapse management. A preemptive treatment option focuses on molecular relapsed patients who have warning signals of imminent HR, including patients with measurable residual disease (MRD) identified by reverse transcription polymerase chain reaction (RT-PCR) or multiparameter flow cytometry, as well as those with decreased donor chimerism in bone marrow (10–12). Recent studies have clearly indicated that treatment starting at molecular relapse is more effective to provide favorable survival, buying time to establish a clinically significant GVL effect in advance of HR (13). In about 20% of AML patients, distinct fusion genes, including *RUNX1-RUNX1T1*, are approachable for MRD monitoring (14–17). It has been confirmed that a significantly shorter leukemia free survival and higher cumulative rate of recurrence were found in patients with persistent MRD detected by positive *RUNX1-RUN1T1* after allo-HSCT (17). Consequently, an increase of MRD maker *RUNX1-RUN1T1* is verified to identify patients at risk for HR and thus guide early prophylactic interventions to prevent relapse and improve the clinical outcomes.

Patients with MRD persistence after allo-HSCT may respond to treatments aiming to restore or augment the GVL effect. Besides pharmacological anti-leukemic approaches, cellular immunotherapy strategies stand for the second backbone for the prevention and treatment of AML recurrence after allo-HSCT by interfering with the immune microenvironment and optimizing the GVL effect (11, 18). Immune escape from the GVL effect contributes to relapse after allo-HSCT. Cytotoxic T-lymphocyte-associated protein 4 (CTLA-4) and programmed cell death protein 1 (PD-1) are the well-known immune checkpoints leading to tumor escape (19, 20). Although triggering severe graft-vs-host disease (GVHD) has limited their widespread application, immune checkpoint inhibitors (ICIs) targeting CTLA-4 or PD-1 have been effectively applied in the context of allo-HSCT to enhance the GVL effect and bring objective responses for recurrent hematological malignancies such as Hodgkin lymphoma, non-Hodgkin lymphoma and AML (21–25). Moreover, to safely promote the GVL effect in this setting, hypomethylating agents (HMAs) such as decitabine and azacitidine are often conferred together with other diverse immunotherapies due to their dual effects of direct cytotoxicity and immune regulation. Recent studies have demonstrated the potential of HMAs to augment the anti-tumor activity and to possibly mitigate immune-mediated toxicities of PD-1 blockade with respect to the relapse of Hodgkin lymphoma or AML (26–28). In view of these promising preliminary clinical data, an anti-PD-1 antibody in combination with azacitidine was applied in a post-HSCT patient in this report. Furthermore, in order to mitigate possible risk of GVHD, a low-dose regimen was also used.

Additionally, recent studies have shown that the detection of severely depleted PD-1-expressing T-cells shortly following allo-HSCT is predictive of disease recurrence and is associated with worse survival (29–32). These findings indicate that ICIs intervention could be possibly used to prevent recurrence in leukemia patients with early evidence of T cell failure after allo-HSCT. Up to now, there is not yet a role for the preemptive or prophylactic administration of ICIs to prevent AML relapse after allo-HSCT. In the present study, we report for the first time an AML patient with *RUNX1-RUNX1T1* who underwent preemptive treatment with tislelizumab (BeiGene, China), an antihuman PD-1 monoclonal IgG4 antibody, together with azacitidine to avoid HR after allo-HSCT.

## Case Description

**Figure 1:**
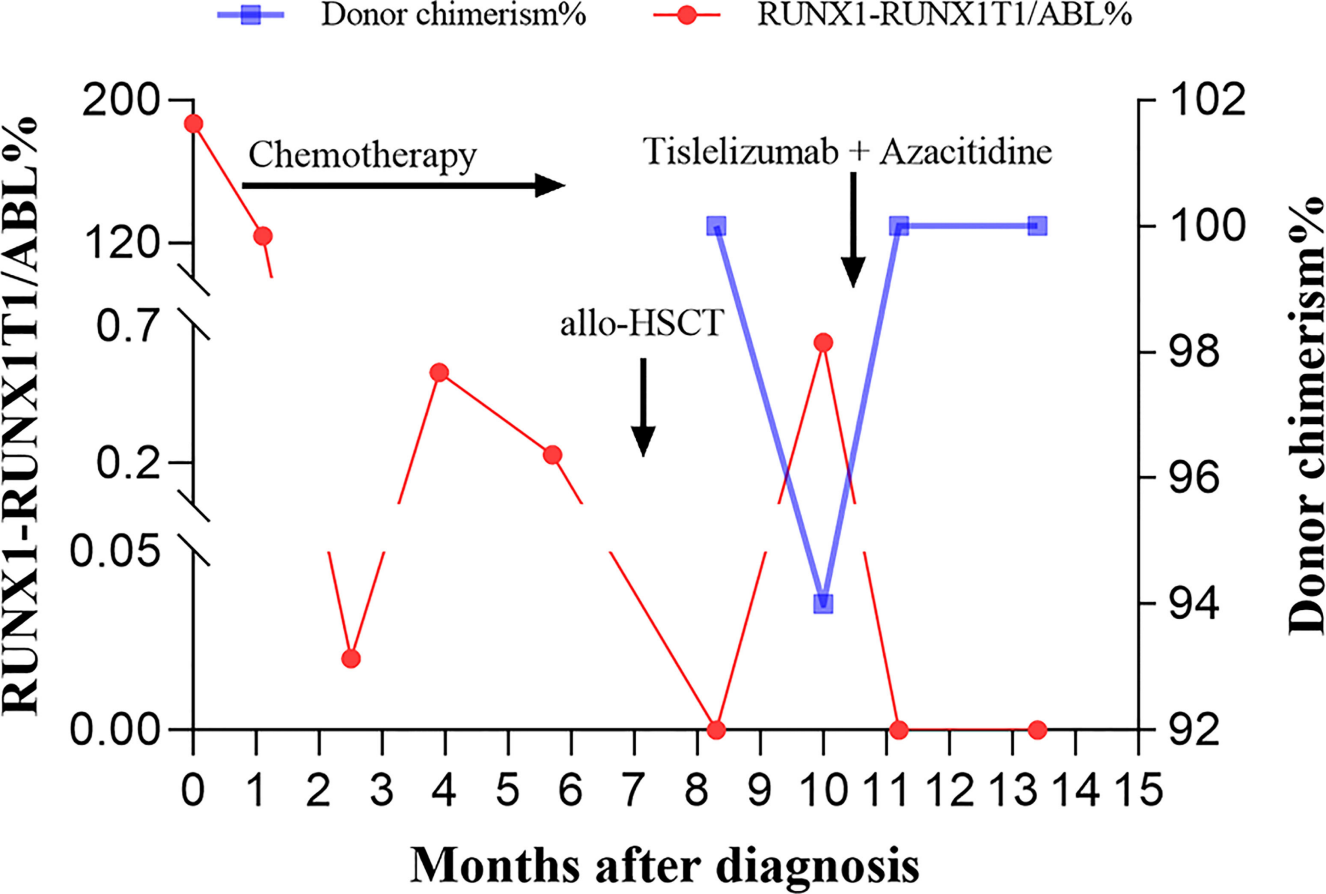
The bone marrow *RUNX1-RUNX1T1/ABL%* and donor chimerism levels at indicated time points following diagnosis are shown. Arrows indicate the time to initiate the chemotherapy, allogeneic hematopoietic stem cell transplantation (allo-HSCT), as well as the combination treatment with tislelizumab and azacitidine, respectively.

**Figure 2:**
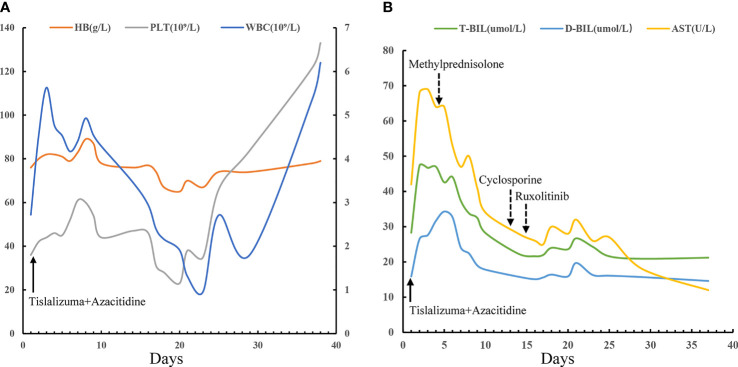
Hematologic responses and changes of liver function following the combination therapy with tislelizumab and azacitidine. 100 mg of tislelizumab was administered on day 1 (solid arrow) and 100 mg of azacitidine was administered on days 1-7. **(A)** Continuous complete blood counts following the combination therapy with tislelizumab and azacitidine showing obvious improvement in WBC and PLT. PLT and HB are provided on the left y-axis, while WBC are on the right y-axis. **(B)** AST, T-BIL, and D-BIL as biomarkers of liver graft-versus-host disease and responses to immunosuppressive therapies (dotted arrow). WBC, white blood cells; PLT, platelets; HB, hemoglobin; AST, aspartate aminotransferase; T-BIL, total bilirubin; D-BIL, direct bilirubin.

A 42-year-old man was originally diagnosed with favorable risk AML in 2019. Cytogenetics revealed a translocation t (8;21) (q22; q22.1) generating a leukemia-specific fusion gene product, *RUNX1-RUNX1T1* (186.81%, %copies/*ABL*). In addition, next-generation sequencing did not detect any gene mutation. *RUNX1/RUNX1T1* mRNA levels were monitored throughout the course of disease using RT-PCR (**Figure 1**). After a cycle of induction chemotherapy with idarubicin and cytarabine, the patient achieved a complete remission (CR), while the expression of *RUNX1/RUNX1T1* still maintained a high level (124.01%, %copies/*ABL*). Then, the patient received three cycles of consolidation chemotherapy with high dose cytarabine contributing to continuous CR. However, molecular remission was not obtained with an MRD determined by the positive *RUNX1/RUNX1T1* (0.23%, %copies/*ABL*). Therefore, allo-HSCT was carried out as a potential curative approach for this high-risk patient. In December 2019, the patient received granulocyte-colony stimulating factor-mobilized peripheral blood stem cell (CD34^+^ 7.01×10^6^ cells/kg) from his human leukocyte antigen (HLA) identical brother, with fludarabine (30mg/m2/day, -6 to -2 days), busulfan (3.2mg/kg/day, -5 to -3 days) and cytarabine (2g/m2/day, -6 to -2 days) as conditioning regimen and short-term methotrexate and cyclosporine as prophylactic agents of GVHD. His platelets and neutrophils were implanted on day +14 and +12, respectively. One month after allo-HSCT, the patient successfully obtained CR with negative MRD (**Figure 1**). At our transplantation center, bone marrow aspirations and monitoring of MRD are always performed at one-month intervals. Unfortunately, since the novel coronavirus (COVID-19) epidemic broke out in Wuhan in December 2019 and spread across the country, the patient was not regularly followed up after transplantation until the whole blood count revealed deterioration. On day +81, he had a molecular relapse with an MRD depicted by *RUNX1-RUN1T1*-positivity (0.64%, %copies/*ABL*) as well as mixed donor chimerism (94%) detected by multiplex short tandem repeat polymerase chain reaction (STR-PCR) in his bone marrow (**Figure 1**). The immunosuppressor was immediately withdrawn, but failed to induce GVHD. Withdrawal of immunosuppression (WIS) a simple and effective way to reinduce or augment clinical GVL effect. The GVL effect is typically coexpressed with GVHD, given that most patients who respond to WIS suffer from GVHD. Unfortunately, GVHD was not induced after WIS in this patient. Meanwhile, donor lymphocyte infusion (DLI) was not available due to COVID-19. Therefore, PD-1 inhibitor was potentially used to induce the GVL effect in this patient. The patient was informed of the benefits and potential risks of tislelizumab combined with azacitidine, while tislelizumab was administered at the lowest practical dose to mitigate GVHD. On day +95, with no signs of GVHD and an excellent eastern cooperative oncology group performance status (ECOG PS) of 1, the patient thus was treated with 100 mg of tislelizumab on day 1 and 100 mg of azacitidine on days 1-7. Following administration of tislelizumab in combination with azacitidine, the patient developed marked pancytopenia, which was markedly improved two weeks later (**Figure 2A**). On day +117, CR was confirmed continuously and the MRD marker *RUNX1/RUNX1T1* eventually turned negative (**Figure 1**). At the same time, the patient regained complete donor chimerism in his bone marrow (**Figure 1**).

**Figure 3:**
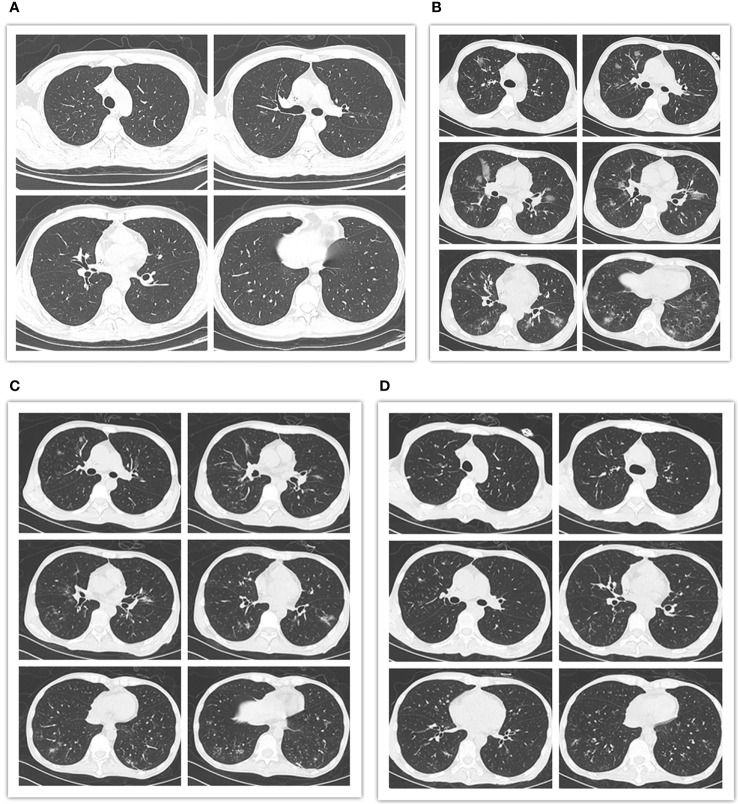
Pre- and post-treatment chest computed tomography (CT) scans. **(A)** CT images obtained 3 days prior to tislelizumab and azacitidine therapy showing no signs of infection. **(B)** CT images obtained 3 days after initiation of the combination therapy revealing bilateral new ground glass opacities. **(C)** CT images obtained 5 days after the administration of methylprednisolone showing obvious improvement in the bilateral lobe lesions. **(D)** CT images obtained 1 month following initiation of methylprednisolone therapy. The bilateral lobe lesions were completely absorbed and disappeared.

However, despite dose reduction, tislelizumab still induced immune related adverse events (irAEs) or/and GVHD in this patient. The patient successively developed fever, cough, liver dysfunction, diarrhea and hematuria. Firstly, the patient occurred high fever on the first day of treatment, with the onset of cough and dyspnea in the next 2 days, accompanied by a significant increase of C-reactive protein (CRP) (102 mg/L). The effect of multiple antibiotic treatments was limited. Then the chest computed tomography (CT) scan revealed bilateral new ground glass opacities (**Figure 3B**), whereas no signs of infection were found by CT three days before treatment (**Figure 3A**). Under the circumstances, the diagnosis of grade 2 immunotherapy-related pneumonitis was proposed depending on the medical history, clinical manifestation as well as CT imaging. Methylprednisolone, 1mg/kg/day, was prescribed and the patient’s condition was immediately improved (CRP, 7.23mg/L) three days later. Importantly, CT showed obvious improvement in the bilateral lobe lesions that were completely absorbed and disappeared one month later (**Figures 3C, D**). Simultaneously, these symptoms were accompanied by diarrhea and a rise in liver function test indexes with total bilirubin (T-BIL) going up to 47.3umol/L and aspartate transaminase (AST) rising up to 69 U/L (**Figure 2B**). Repeated stool etiology examination showed no definite infection. Meanwhile, gastrointestinal and liver biopsies were not performed. After above methylprednisolone treatment, the symptoms were relived and liver function markers were significantly improved (**Figure 2B**). The response to methylprednisolone confirmed the clinical diagnosis of grade II acute GVHD involving the gastrointestinal tract and liver. Subsequently, the patient developed hematuria and dysuria 10 days after receiving tislelizumab treatment. The CT scan demonstrated that there were hydronephrosis, hydroureter and inflammatory changes. Urine etiological tests were negative and renal function was normal. A diagnosis of irAEs involving the urinary system was hypothesized, based on history, clinical manifestations, and imaging. Methylprednisolone was tapered and cyclosporine (minimum concentration at 150 ng/ml) along with ruxolitinib (10 mg/day) were started. The patient indeed had a response and the clinical symptoms of hematuria and dysuria disappeared after 2 weeks. In this patient, blocking of PD-1 pathway caused moderate GVHD or/and irAEs of the liver, lung, lower digestive tract and urinary system, which were responsive to steroid and other immunosuppressant. Of note, the patient had a generalized inflammatory reaction with high markers including IL-2, IL-6, IL-17A, and TNF-α after tislelizumab treatment. Similarly, a recent report has demonstrated that checkpoint inhibition is related to a proinflammatory immune response with the upregulation of inflammatory factors, which may be associated with GVHD and irAEs (33, 34).

Finally, response was ongoing 60 days after tislelizumab therapy with negative MRD (**Figure 1**), and there were no symptoms of GVHD or irAEs. Unfortunately, the patient ultimately died of asphyxiation after accidental inhalation of drugs three months following tislelizumab treatment.

## Discussion

Loss of GVL effect is generally accountable to the relapse after allo-HSCT, which leads to tumor cells escaping from allogeneic immune response (12, 35). With the clinical breakthrough of ICIs, boosting the GVL effect with ICIs post-allo-HSCT has become an appealing concept to treat relapse and improve prognosis. In this report, a role for the preemptive intervention with ICIs to prevent AML relapse after allo-HSCT was explored. Tislelizumab in combination with azacitidine was effective for molecular relapsed AML following allo-HSCT. The patient eventually achieved sustained CR with negative MRD.

In the post-HSCT relapse management, a preemptive therapy approach to prevent disease progression is more attractive and effective. Preemptive treatment with DLI is one of the most effective strategies to restore the GVL effect after allo-HSCT, whereas it is not actually available for all patients because of donor decline and severe GVHD (36, 37). New methods are urgently needed to reinvigorate the GVL effect in these patients. With detailed studies on the mechanisms of AML recurrence after allo-HSCT, a better understanding of the GVL response is beginning to emerge. In addition to HLA loss and HLA class II downregulation, the increased expression of immune checkpoints is another important arm of tumor immune escape, which can be treated by ICIs therapy (38). In this case, patients are in a distinctive position to benefit from ICIs therapy as compared with DLI. Additionally, DLI acting on relapsed patients is directly mediated by the cytotoxicity of effector T cells from the donor-derived products, whereas ICIs work by activating functional cells that already reside in the tumor microenvironment. Accordingly, ICIs agents that could reverse T-cells failure properties might be especially effective in patients with recurrence following allo-HSCT. Clinically, anti-PD-1 therapy with nivolumab induced remission in a recurrent Hodgkin lymphoma patient after allo-HSCT who was refractory to DLI (39). This finding also suggests that PD-1 blockade may be more effective in activating the GVL effect through distinct pathways. Importantly, a recent study has shown that the detection of increased PD-1-expressing T cells shortly after transplantation was related to poor survival (29). Furthermore, Kong and colleagues found that the PD-1-high TIM3+ T cells with exhaustion signatures appeared ahead of clinical diagnosis of disease recurrence, which indicated that they may possess predictive potential (30). These findings suggest that preemptive therapy with ICIs could be possibly used to prevent recurrence in patients with early detection of exhausting T cell after allo-HSCT. Indeed, preemptive treatment with PD-1 blockade and azacitidine showed an encouraging response with respect to the molecular relapse of AML in our case. In conclusion, anti-PD-1 treatment may open a novel option for immune modulation to prevent HR following allo-HSCT.

It is understandable that ICIs may potentially trigger uncontrollable immune breakthrough events, in particular severe GVHD, in the post-transplant setting (20, 35, 40). Therefore, the major obstacle with ICIs is to control the severity of GVHD while maintaining the GVL effect. Various pre-transplantation and post-transplantation factors exert a significant role in the progression of GVHD, such as the intensity of conditioning regimen, the dose and timing options of ICIs, as well as GVHD prophylaxis and treatment. There has a report that provides recommendations for the dosage and timing of PD-1 blockade, along with the management of GVHD-related adverse events in the context of allo-HSCT in Hodgkin lymphoma (41). Currently, emerging interest has been focused on the potential to use ICIs in the setting of myeloid malignancies with relapse after allo-HSCT and many strategies to ameliorate GVHD are being explored. In a multicenter, phase I clinical study using nivolumab for recurrent hematologic malignancies after allo-HSCT, the ECOG PS was found to be the only factor associated with the development of irAEs (21). That was the reason why we preferred to use PD-1 inhibition for patients with molecular relapse, who generally have lower ECOG PS with better tolerability when compared with patients of HR. In addition, it is widely accepted that first administration with low doses of PD-1 blockade after transplant may be a safer strategy. Increasing studies have suggested that lower doses of PD-1 blockade possibly bring obvious responses without inciting clinically significant adverse events (21, 24, 41–43). It is worth noting that further dose exploration research is of high importance for different underlying malignancies in the future. The dose of tislelizumab in our case was substantially lower than was typically used outside the allo-HSCT setting, which appeared to be feasible and safe despite the observation of GVHD and irAEs. The optimal dose of PD-1 pathway blocking antibody to unleash the GVL effect without inducing significant GVHD and irAEs needs further clinical investigation.

Moreover, the time to ICIs initiation is controversial and remains to be explored. A recent study has showed that a shorter interval between allo-HSCT and initial PD-1 blockade administration was related to an increased risk of attendant GVHD, although there was no statistically significant difference (21). Similarly, two studies observed that GVHD developed more frequently in lymphoma patients after allo-HSCT who received PD-1 blockade at an earlier stage (23, 24). Although the definition of “earlier” remains unclear, a recent study encourages the initiation time of PD-1 blockade beyond 180 days after allo-HSCT for Hodgkin lymphoma (41). However, another study found that GVHD occurrence was independent from the initiation time of ICIs therapy after allo-HSCT in diseases other than Hodgkin lymphoma (44). In this report, the time from transplant to the anti-PD1 treatment was 95 days, which was significantly shorter than the recommended time. The development of GVHD and irAEs was indeed observed, but was well controlled. Since many patients develop HR within a few months of transplantation, treatments aiming at lessening the risk of recurrence should be initiated at a relatively earlier phase of post-transplantation; and the inability to use ICIs early after allo-HSCT may limit the eligible population. Therefore, we suggest that early initiation of ICIs therapy after allo-HSCT is feasible and deserves attention. Besides, a recent study has also showed that irAEs directly attributed to ICIs therapy may be alleviated in patients with AML/myelodysplastic syndrome (MDS) who receive post-transplantation cyclophosphamide as GVHD prophylaxis (45). Currently, we have not changed our conditioning regimen in advance considering the possibility of disease relapse and ICIs therapy. However, this finding is attractive and should be taken into account particularly when designing clinical trials to evaluate ICIs after allo-HSCT.

Furthermore, alternative approaches to safely promote the GVL effect in post-transplant setting could take advantage of the beneficial effects of HMAs. One such method combines DLI with HMAs, and has revealed efficacy especially in patients with molecular and/or late relapses in myeloid malignancies (46, 47). Recent studies also observed the potential of HMAs to augment the anti-tumor activity and to possibly mitigate immune-mediated toxicities of anti-PD-1 antibodies with respect to the recurrence of Hodgkin lymphoma and AML/MDS (27, 28), suggesting that the combination therapy could be worthy of further exploration for patients who relapse after allo-HSCT. In terms of mechanism, the combination therapy of HMAs and PD-1 blockade could weaken their respective disadvantages and give full play to their respective strengths. On the one hand, the upregulation of immune checkpoint molecules may be a crucial mechanism accounting for azacitidine resistance (27, 48, 49), thus the addition of PD-1 blockade to azacitidine may produce more effective responses in HMAs-resistant patients. On the other hand, the evasion mechanisms of ICIs treatment involve reduced levels of tumor antigen, downregulation of major histocompatibility complex, as well as loss of costimulatory ligand expression, which can be eliminated by HMAs with powerful antitumor immunity (26). What’s more, azacitidine mitigates GVHD while maintaining a robust GVL effect in murine transplant models as well as human clinical trials (28, 50, 51). Based on these promising preliminary clinical results, an anti-PD-1 antibody combined with azacitidine was applied in a post-HSCT patient in this study and induced a remarkable and fast response. Therefore, the potential synergistic effect of anti-PD-1 antibodies + azacitidine combination therapy should be urgently investigated further in the post-transplantation setting.

We are just beginning to touch the tip of the iceberg in the understanding of whether ICIs therapy should be used after allo-HSCT, when they should be given, which drugs to choose, and what doses to apply. Nonetheless, monitoring and close clinical assessment of treatment-related adverse events are extremely important for patients receiving ICIs therapy after allo-HSCT (41). Although the ideal treatment of GVHD secondary to PD-1 inhibition remains unclear, early initiation of immunosuppressant may be helpful to GVHD treatment. Once GVHD is suspected, we recommend immediate discontinuation of treatment and prompt initiation of systemic corticosteroids, along with diagnostic procedures. If patients develop steroids-refractory GVHD, alternative immunosuppressive therapies must be quickly initiated. In the present report, GVHD and irAEs were well controlled with steroids, cyclosporine and ruxolitinib when timely applied.

Although DLI has been the principal strategy to address post-HSCT recurrence by augmenting the GVL effect for many years, anti-PD1 antibodies can be modulated more easily than donor-derived cellular products to selectively induce the GVL effect without triggering severe GVHD. Therefore, we believe that anti-PD-1 antibodies in combination with HMAs such as azacitidine is a suitable option for preventing and treating AML relapsing after allo-HSCT. Furthermore, on the premise of safety, we would prefer to apply ICIs therapy in a prophylactic or preemptive manner instead of waiting for complete disease recurrence, thus improve the prognosis of transplant recipients.

## Conclusions

To the best of our knowledge, this case is the first one to report an objective response with some durability to PD-1 inhibition in combination with azacitidine, complicated by the moderate GVHD and irAEs of the lung, lower digestive tract, liver, and urinary system in the setting of a molecular relapsed AML patient after allo-HSCT. The application of ICIs in MRD-positive patients might also be a promising approach for cellular immunotherapy in the future to overcome the risk of HR in the context of post-transplant, but safer clinical application principles need to be established.

## Data Availability Statement

The original contributions presented in the study are included in the article/supplementary material. Further inquiries can be directed to the corresponding authors.

## Ethics Statement

The studies involving human participants were reviewed and approved by the ethical committee of Tongji Medical College, Huazhong University of Science and Technology. The patients/participants provided their written informed consent to participate in this study. Written informed consent was obtained from the individual(s) for the publication of any potentially identifiable images or data included in this article.

## Author Contributions

HY and YY obtained and analyzed the clinical data. YT and ZZ made the figures. The authors all contributed to caring for the patient, editing the figures, and writing and editing the manuscript. All authors contributed to the article and approved the submitted version.

## Funding

This work was supported by the National Natural Science Foundation of China (grant number 81500168).

## Conflict of Interest

The authors declare that the research was conducted in the absence of any commercial or financial relationships that could be construed as a potential conflict of interest.

## Publisher’s Note

All claims expressed in this article are solely those of the authors and do not necessarily represent those of their affiliated organizations, or those of the publisher, the editors and the reviewers. Any product that may be evaluated in this article, or claim that may be made by its manufacturer, is not guaranteed or endorsed by the publisher.
